# Differential role of the menthol-binding residue Y745 in the antagonism of thermally gated TRPM8 channels

**DOI:** 10.1186/1744-8069-5-62

**Published:** 2009-11-03

**Authors:** Annika Malkia, María Pertusa, Gregorio Fernández-Ballester, Antonio Ferrer-Montiel, Félix Viana

**Affiliations:** 1 Instituto de Neurociencias de Alicante, Universidad Miguel Hernández-CSIC, 03550 San Juan de Alicante, Spain; 2 Instituto de Biología Molecular y Celular, Universidad Miguel Hernández, 03202 Elche, Spain

## Abstract

**Background:**

TRPM8 is a non-selective cation channel that belongs to the melastatin subfamily of the transient receptor potential (TRP) ion channels. TRPM8 is activated by voltage, cold and cooling compounds such as menthol. Despite its essential role for cold temperature sensing in mammals, the pharmacology of TRPM8 is still in its infancy. Recently, tyrosine 745 (Y745) was identified as a critical residue for menthol sensitivity of the channel. In this report, we study the effect of mutating this residue on the action of several known TRPM8 antagonists: BCTC, capsazepine, SKF96365, and clotrimazole as well as two new inhibitor candidates, econazole and imidazole.

**Results:**

We show that Y745 at the menthol binding site is critical for inhibition mediated by SKF96365 of cold- and voltage-activated TRPM8 currents. In contrast, the inhibition by other antagonists was unaffected by the mutation (BCTC) or only partially reduced (capsazepine, clotrimazole, econazole), suggesting that additional binding sites exist on the TRPM8 channel from where the inhibitors exert their negative modulation. Indeed, a molecular docking model implies that menthol and SKF96365 interact readily with Y745, while BCTC is unable to bind to this residue.

**Conclusion:**

In summary, we identify structural elements on the TRPM8 channel that are critical for the action of channel antagonists, providing valuable information for the future design of new, specific modulator compounds.

## Background

TRPM8 is a non-selective cation channel of the TRP family that is activated by mild cold temperatures and cooling compounds such as menthol, eucalyptol and icilin [1,2]. Like several other TRP channels, TRPM8 is also gated by voltage [3-6]. The voltage dependence of TRPM8 is characterised by a strong outward rectification at depolarized transmembrane potentials, and a rapid and potential-dependent closure at negative membrane potentials. Cooling and menthol application shift the activation curve of TRPM8 towards more negative potentials, thus increasing the probability of channel openings, boosting inward currents at physiological membrane potentials [6]. Endogenous factors such as phospholipase A_2 _products [7,8], endocannabinoids [9] and PIP_2 _[10-12] also participate in channel regulation.

TRPM8 is expressed in a subset of small diameter primary sensory neurons and their peripheral terminals [13,1,2]. In addition to its well characterized and critical role in the activation of low threshold thermoreceptors, responsible for the sensations of innocuous cold [14-16], other evidence indicates the possible involvement of TRPM8 channels in normal noxious cold sensations and cold allodynia [reviewed by [17]]. Notably, in an animal model of neuropathic pain, cold allodynia is significantly attenuated by capsazepine, a TRPM8 blocker [18], and mice lacking TRPM8 show reduced responses in nerve injury induced models of cold-allodynia [15,16]. Also, sensory fibers with high threshold cold-evoked responses are difficult to record from in these mice [14]. Moreover, TRPM8-positive fibers are prominent in peripheral territories with marked noxious responses to cold [19]. Many neurons responding to TRPM8 agonists are also activated by capsaicin, a marker of nociceptors [20-22]. These new findings stress the potential use of TRPM8 modulators in the therapeutic management of cold-evoked pain, a characteristic symptom in some patients with neuropathic pain [23].

Despite its fundamental role in many aspects of cold temperature transduction in mammals, the pharmacology of TRPM8 is still largely unexplored. Only a few studies have so far been dedicated to TRPM8 channel inhibitors and their mechanisms of action [24-30]. Recently, Malkia et al. [26] showed that several antagonist compounds, including BCTC and SKF96365, act as negative allosteric modulators of channel gating, shifting the voltage activation of TRPM8 towards more positive potentials, suppressing the depolarizing effects of temperature and chemical agonists [25,26]. SKF96365 is a non-specific blocker of various calcium-permeable channels, including receptor-operated channels [31]. BCTC was originally introduced as a highly potent and specific antagonist of the heat-activated vanilloid receptor TRPV1 [32]. However, later studies showed that it also inhibits the TRPM8 channel, as does another TRPV1 blocker, capsazepine [24,26,29]. These two antagonists bind competitively at the vanilloid binding pocket of the TRPV1 channel, governed by residues in the intracellular parts of the putative transmembrane domains 2, 3 and 4 [33,34].

Recently, during a massive random mutagenesis screen, tyrosine 745, located in the middle of putative transmembrane segment 2, was identified as a crucial residue for the menthol sensitivity of mouse TRPM8 [35]. The generated TRPM8-Y745H mutant channel was insensitive to menthol, but retained the responsiveness to cold and voltage exhibited by the wild-type channel. Because of the significant parallels between TRPM8 and TRPV1 pharmacology [24,29], we decided to study the effect of the Y745H mutation on the activity of the best characterized TRPM8 antagonists: BCTC, capsazepine, SKF96365 and clotrimazole, as well as of two new inhibitor candidates: econazole and imidazole. We identify, for the first time, structural elements on the TRPM8 protein that are critical for channel antagonism, and demonstrate an important difference in the way antagonists interact with the menthol binding site of TRPM8.

## Results

### Expression and functional phenotype of the TRPM8-Y745H mutant

**Figure 1:**
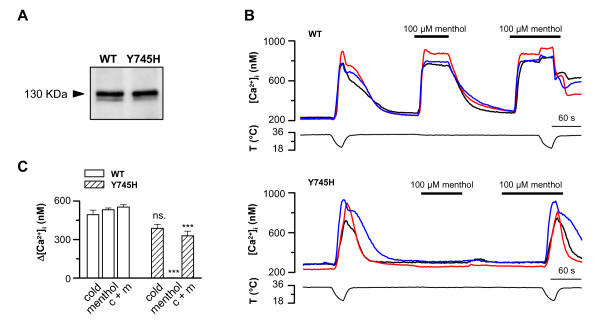
**The TRPM8-Y745H mutant is insensitive to menthol, but retains cold sensitivity**. *A*, Western blot where the lanes represent lysates of HEK293 cells transfected with TRPM8-wt and TRPM8-Y745H. *B*, Representative traces showing calcium imaging experiments of HEK293 cells expressing TRPM8-wt or TRPM8-Y745H. Note that only the cells expressing TRPM8-wt respond to menthol. *C*, Summary histogram of experiments seen in B. Intracellular calcium increases were compared using repeated-measures 2-way ANOVA in combination with Bonferroni's post test with respect to the effect of the mutation on each condition: *** p < 0.001; n = 14 (TRPM8-wt) and n = 19 (TRPM8-Y745H).

The correct expression of the TRPM8-Y745H mutant was first verified by Western blot analysis of lysates from HEK293 cells transfected with the TRPM8-wt and TRPM8-Y745H plasmids. The TRPM8 antibody strongly detected both the wild-type and the mutant constructs at the expected size of 130 kDa (Figure 1A).

**Figure 2:**
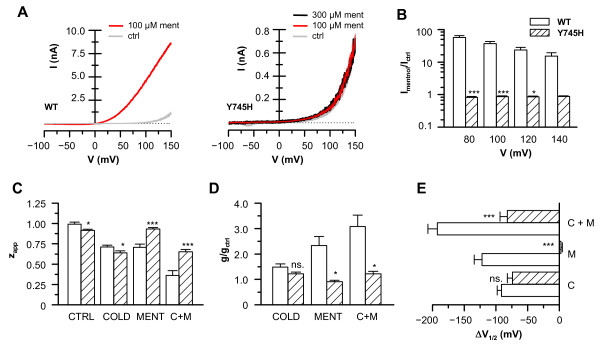
**Electrophysiological characterization of TRPM8-Y745H mutant sensitivity to menthol, cold and voltage**. *A*, Whole-cell current-voltage relationships of TRPM8-wt and TRPM8-Y745H expressing HEK293 cells in control and menthol-containing solutions at 33°C. Note the different current scale. *B*, Summary histogram of experiments seen in A showing menthol-induced whole-cell currents at various potentials, normalized with the current in control conditions. Note the logarithmic current scale. The responses of TRPM8-wt *vs*. Y745H were compared with repeated-measures 2-way ANOVA in combination with Bonferroni's post test with respect to the effect of the mutation at each potential: *** p < 0.001; * p < 0.05; n = 7 (TRPM8-wt) and n = 13 (TRPM8-Y745H). *C*-*E*, Parameters obtained from fits of I-V data of experiments such as the ones showed in A to equation (i), n = 4-19. *C*, Average values of the apparent gating charge in control (33°C), menthol (100 μM), cooling (20°C) and cooling + menthol, of the TRPM8-wt vs. TRPM8-Y745H. *D*, Average maximum conductance (g) of cells expressing TRPM8-wt and TRPM8-Y745H during cooling and menthol application. The data are normalized with the value of g of the same cells in control solution at 33°C. *E*, Agonist-induced shifts of the midpoint of voltage activation (V_1/2_) of TRPM8-wt *vs*. TRPM8-Y745H. The data are represented with respect to the value of V_1/2 _in control solution at 33°C. In panels C-E, statistical significance was assessed with Student's unpaired *t*-test: *** p < 0.001; ** p < 0.01; * p < 0.05.

Next, the functionality of the TRPM8-Y745H mutant was investigated employing the calcium imaging technique. All GFP expressing cells exhibited robust responses to cooling of the bath solution, indicative of the presence of a cold-sensitive TRPM8 variant (Fig. 1B-C). However, only in TRPM8-wt transfected cells did 100 μM menthol increase the intracellular calcium levels when applied at 33°C or during cooling. The lack of menthol responses of the Y745H mutant was also confirmed with whole-cell electrophysiological recordings, which showed that the Y745H mutant channel was completely insensitive to menthol at all potentials (Fig. 2A-B). Both, the calcium imaging and the electrophysiological recordings confirmed the critical role of Y745 in menthol-dependent activation of TRPM8 [35].

Next, to investigate whether the mutation at the Y745 residue affected the voltage-dependent channel gating we performed a detailed analysis of the I-V curves obtained from -100/+150 mV voltage ramps by fitting them to equation (i) under different agonist conditions. As seen in Figures 2C-E, no differences in the cold-induced increase in maximum conductance, g_cold_/g_ctrl_, or shift in midpoint voltage of activation, ΔV_1/2_, were observed between the wild-type channel and the Y745H mutant, whereas a clear difference was present when menthol was used as the agonist. The absolute value of V_1/2 _during cooling was likewise similar in the wild-type and mutant channels (wt: 83 ± 6 mV, n = 19 *vs*. Y745H: 76 ± 11 mV, n = 14; unpaired *t*-test; p = 0.56).

The apparent gating charge, z_app_, was slightly lower in the Y745H mutant compared with the wild-type during control and cooling conditions. While menthol decreased the gating charge of the wild-type channel, the mutant channel gating charge was unaffected. When we compared the I-V parameters of the Y745H mutant during applications of cold and cold plus menthol, no difference was observed indicating that the Y745 residue also perfectly accounts for any sensitizing effect that menthol has on the cold response (paired *t*-test; p > 0.05; n = 6).

### Sensitivity of the TRPM8-Y745H mutant to BCTC, SKF96365, capsazepine and clotrimazole

**Figure 3:**
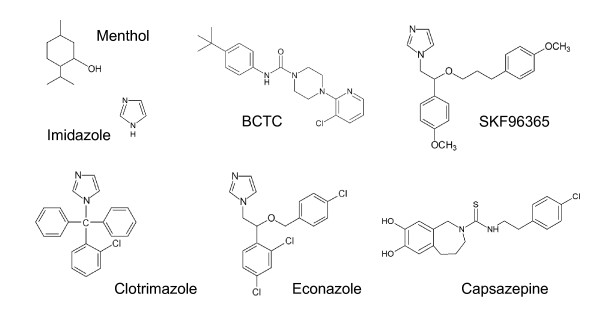
**Structures of the various TRPM8 antagonists and menthol**. Chemical structures of the various antagonists tested at the TRPM8-wt and Y745H mutant channels. The structure of menthol is shown for comparison.

**Figure 4:**
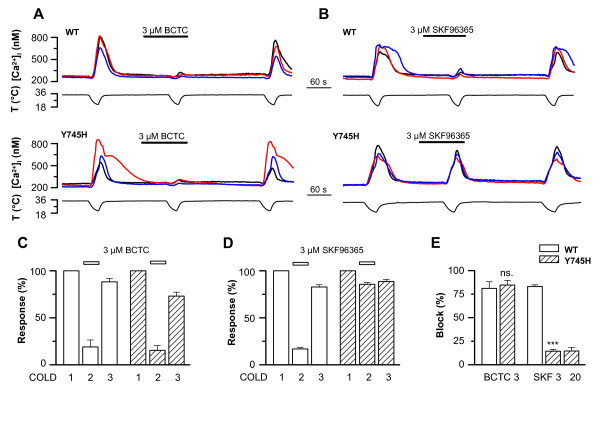
**Differential effect of the Y745H mutation on the antagonism of TRPM8 by BCTC and SKF96365**. *A*-*B*, Cold-evoked [Ca^2+^]_i _responses in HEK293 cells expressing TRPM8-wt or TRPM8-Y745H channels, showing the inhibition by *A*, 3 μM BCTC, and *B*, 3 μM SKF96365. *C*-*D*, Summary histograms of the [Ca^2+^]_i _responses of TRPM8-wt and TRPM8-Y745H channels to repeated cooling stimuli in the absence and presence of *C*, 3 μM BCTC (n = 9/11 wt/mut); and *D*, 3 μM SKF96365 (SKF; n = 33/47). Note the reversible nature of the inhibition. *E*, Comparison of block of TRPM8-wt *vs*. TRPM8-Y745H by BCTC and SKF96365. In panels C-E, intracellular calcium increases were normalized to the first cold application in control solution. In panel E, the block of each antagonist condition was compared between TRPM8-wt and -Y745H using Student's unpaired *t*-test: *** p < 0.001; ns. = not significant. The block by 20 μM SKF96365 over the Y745H mutant is included to show the complete lack of inhibition.

We next proceeded to investigate the ability of several known TRPM8 antagonists to block the TRPM8-Y745H mutant channel during activation by cold. In Figure 3 and Table 1, the structures of the blockers and their half maximal inhibitory concentrations at the wild-type channel are given. Dose-inhibition curves of BCTC and SKF96365 at the cooling-activated wild-type channel have been published previously [26]. Calcium imaging experiments revealed that the mutation affected the ability of the antagonists to inhibit TRPM8 activity to a varying extent. While BCTC maintained its full and reversible blocking potential when tested on the Y745H mutant (Figures 4A, C, E), SKF96365, applied at the saturating dose of 3 μM, exhibited a nearly complete loss of effect (Figures 4B, D, E). To determine whether the observed lack of block at the mutant channel was due to a shift in the dose-response curve of SKF96365, we also tested a super saturating concentration of the drug. However, 20 μM SKF96365 (n = 13) was unable to increase block of the cold-evoked response compared with 3 μM of the antagonist (Figure 4E), confirming the complete insensitivity of the Y745H mutant to this compound.

**Table 1:** Summary of inhibition of the TRPM8-wt channel.

Compound	IC_50 _cold (μM)	IC_50 _menthol (μM)
BCTC	0.68 ± 0.06^*a *(CI)^ 0.54 ± 0.04^*a *(EP)^	0.47 ± 0.01^*b *(CI)^ 0.34 ± 0.04^*b *(EP)^

SKF96365	1.0 ± 0.2^*a *(CI)^ 0.8 ± 0.1^*a *(EP)^	3 ± 1^*b *(CI)^

Capsazepine	12 ± 2^*d *(CI)^	18 ± 1^*c *(FLIPR)^

Clotrimazole	8 ± 1^*d *(CI)^	1.2^*e *(EP)^

Econazole	0.42 ± 0.07^*d *(CI)^	N/A

Imidazole	>> 1000	N/A

Inhibitory potency of various blockers at the cold- or 100 μM menthol-activated TRPM8-wt channel as measured by calcium imaging (CI), a fluorometric imaging plate reader assay (FLIPR) or electrophysiology (EP). Data are from: ^*a*^[26]; ^*b*^modified from [25]; ^*c*^[24]; ^*d*^this work; ^*e*^[27]. Whole-cell patch-clamp data are given at +80 mV, except for ^*e*^, where the block was estimated at +50 mV.

**Figure 5:**
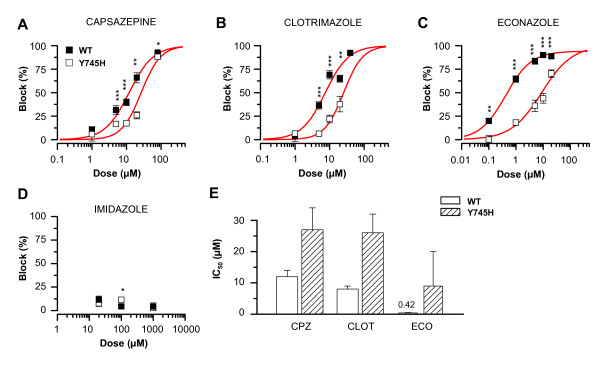
**Variable inhibition of the Y745H mutant channel by capsazepine, clotrimazole, econazole and imidazole**. *A*-*D*, Dose-inhibition curves of various antagonists at the cooling-activated TRPM8-wt and TRPM8-Y745H channels: *A*, capsazepine (n = 17-23/13-29 wt/mut); *B*, clotrimazole (n = 7-17/12-25); *C*, econazole (n = 14-27/9-27); and *D*, imidazole (n = 12-21/13-16). *E*, Comparison of the IC_50 _of block of TRPM8-wt *vs*. TRPM8-Y745H by the above antagonists: capsazepine (CPZ); clotrimazole (CLOT); econazole (ECO). In panels A-D, the red traces represent the fits to the Hill equation. Error bars were used as weights in fitting. At each concentration, the block was compared between TRPM8-wt and -Y745H using Student's unpaired *t*-test, and indicated where significant by: *** p < 0.001; ** p < 0.01; * p < 0.05.

Two other compounds with reported TRPM8 antagonism, capsazepine and clotrimazole, both lost part of their inhibitory potential at the Y745H mutant. As seen in Figures 5A, B, E, the effect of the mutation was to decrease the potency of these compounds compared with the wild-type channel and to shift their dose-inhibition curves towards higher concentrations. Clotrimazole exhibits limited solubility in the extracellular solution used, and obtaining reproducible data above 20 μM was complicated, especially for the Y745H mutant channel where the dose-inhibition curve was in its steepest phase.

In view of the recent discovery of the inhibitory activity of the imidazole derivatives SKF96365 [31] and clotrimazole at the TRPM8 channel [26,27], we decided to investigate the antagonism of two structurally related compounds, the parent compound imidazole and the antimycotic agent econazole. Similarly to clotrimazole, econazole is an effective antagonist of TRPM2 [36], and has also been reported to potently block TRPV5 channels (ECaC1) [37]. We found that econazole was even more potent than clotrimazole at the wild-type TRPM8 channel as measured by calcium imaging (Figures 5C, E), exhibiting an IC_50 _of 0.42 ± 0.07 μM (n = 9-33). Similarly to clotrimazole, the antagonism of econazole was significantly reduced at the Y745H mutant channel (Figure 5C, E). Imidazole, on the other hand, was ineffective at both the TRPM8 wild-type and Y745H mutant channels, at concentrations of 20 μM, 100 μM and 1 mM (Figure 5D).

While the TRPM8-wt channel is known to undergo certain rundown in the experimental conditions used in our calcium imaging recordings [26], the almost complete recovery of the initial cooling response after 3 minutes of washing observed with the majority of the antagonists (e.g. Figure 4) demonstrates the inhibition to be specific to the antagonists applied.

### Electrophysiology confirms the differential effects of the Y745H mutation on BCTC and SKF96365 antagonism

**Figure 6:**
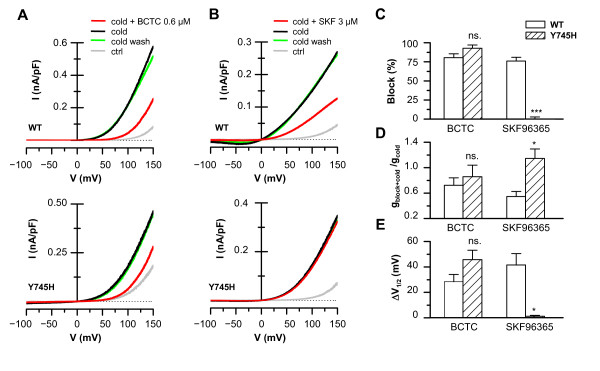
**Electrophysiology confirms the differential effects of the Y745H mutation on BCTC and SKF96365 antagonism**. Whole-cell I-V curves from voltage ramps (-100/+150 mV) of TRPM8-wt and TRPM8-Y745H expressing HEK293 cells during cooling in the presence and absence of *A*, 0.6 μM BCTC; and *B*, 3 μM SKF96365. Wash traces recorded 3 minutes after removal of the antagonist from the bath are included to show the reversible nature of the inhibition. *C*, Summary histogram of experiments depicted in A and B, showing the block of cold-evoked currents by 0.6 μM BCTC and 3 μM SKF96365 at a membrane potential of +80 mV. *D*-*E*, Parameters obtained from fits of I-V data to equation (*i*). *D*, Antagonist-induced change in maximum conductance during cooling in cells expressing TRPM8-wt and TRPM8-Y745H. The data are normalized to the value during cooling in control solution of the same cells, (g_cold+blocker_/g_cold_). *E*, Antagonist-induced shifts of the midpoint of voltage activation (V_1/2_) of TRPM8-wt *vs*. TRPM8-Y745H during cooling. The data are represented with respect to the value of V_1/2 _in the absence of blocker (ΔV_1/2 _= V_1/2, cold+blocker _- V_1/2, cold_). In panels C-E, statistical significance was assessed with Student's unpaired *t*-test, n = 2-6.

In a previous study we showed that calcium imaging provides a reliable estimate of the potency of block of TRPM8 by different antagonists [26]. Nevertheless, in order to assess channel function directly and obtain more detailed information on the mechanism of block, we subsequently employed the whole-cell patch clamp technique. We focused on BCTC and SKF96365 because they showed the largest differences between wild-type and mutant channels. Figure 6A shows I-V curves of the wild-type and Y745H mutant channels during cooling of the divalent-free bath solution in the presence and absence of 0.6 μM BCTC. This lower concentration was chosen so as to obtain more reliable fits to equation (i), and to detect possible dose-response effects not seen with the saturating concentration of 3 μM. Analysis of the I-V curves shows that the Y745H mutant channel exhibits a similar response to BCTC at +80 mV when compared with the wild-type channel (Figure 6C). Furthermore, fits of the curves to equation (i) during activation by cold, allowed us to confirm our previous observation [26] that BCTC inhibits TRPM8 channel activity through a reduction in maximum conductance and a shift of the midpoint activation voltage towards more positive potentials. No significant differences were observed in either of these parameters between the wild-type channel and the Y745H mutant (Figures 6D-E), nor in the apparent gating charge (not shown). The effects were dose-dependent and readily reversible upon wash of the drug (Figure 6A). At 3 μM, BCTC blocked 98% of the cooling-activated TRPM8-Y745H current at +80 mV (not shown).

In close agreement with the calcium imaging experiments, 3 μM SKF96365 exhibited potent antagonism of the wild-type channel, but completely failed to block the cold-evoked activation of the TRPM8-Y745H mutant (Figures 6B-C). Fits to equation (i) revealed that, in contrast to the wild-type channel, in the mutant SKF96365 did not reduce the maximum conductance from the value obtained during cooling in control solution. Likewise the V_1/2 _value of channel activation during cooling did not shift with application of SKF96365 (Figures 6D-E). The apparent gating charge during SKF96365 application was similar in the wild-type and mutant channels (0.73 ± 0.04 vs. 0.63 ± 0.04, respectively, p = 0.25). No dose-dependence was observed as 20 μM SKF96365 also lacked any inhibitory effects (not shown).

**Figure 7:**
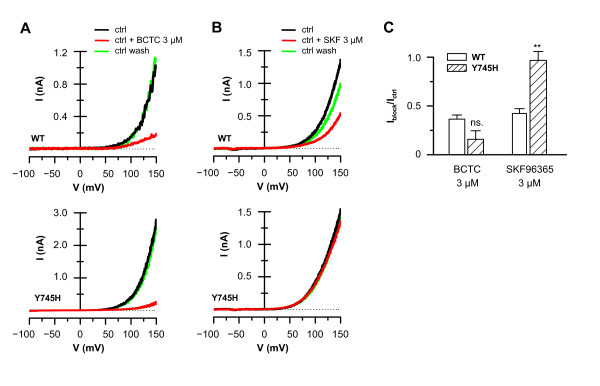
**Differential block of voltage-activated TRPM8-Y745H by BCTC and SKF96365**. Whole-cell I-V curves from voltage ramps (-100/+150 mV) of TRPM8-wt and TRPM8-Y745H expressing HEK293 cells at 33°C in the presence and absence of *A*, 3 μM BCTC; and *B*, 3 μM SKF96365. *C*, Summary histogram of experiments seen in A and B, showing the block of voltage-evoked currents by BCTC and SKF96365 at a membrane potential of +120 mV. Statistical significance was assessed with the unpaired *t*-test: ** p < 0.01, n = 2-5.

The differential effects of the Y745H mutation on BCTC and SKF96365 antagonism during cooling were also observed at 33°C, a condition were TRPM8 is only activated by voltage [6]. In wild-type channels, BCTC and SKF96365 produced a strong inhibition of the voltage-activated current (Figure 7). In contrast, the Y745H mutation fully abrogated the inhibition produced by SKF96365 without affecting the block by BCTC.

### Molecular simulations support the interaction between SKF96365 and Y745

**Figure 8:**
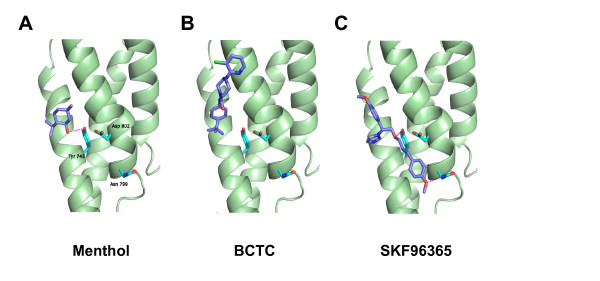
**Molecular modelling of ligand docking into the putative menthol binding site of the TRPM8 receptor**. Parts of the S1-S4 domains of TRPM8 are depicted and residues Y745 in S2 and N799 and D802 in S3 are highlighted. *A*, Menthol makes a hydrogen bond with Y745 (S2), disrupting its interaction with D802 (S3). *B*, BCTC is located externally of Y745 preventing the interaction with this residue. *C*, SKF96365 interacts with Y745 and N799, stabilizing the positions of the S2 and S3 domains. The TRPM8 model was built using the coordinates of Pedretti et al. [38].

To support the experimental data, we probed the docking of menthol, BCTC and SKF96365 at a TRPM8 model protein (Figure 8). Menthol readily interacted with the Y745 binding site, disrupting the potentially stabilizing interactions between this S2 residue and the D802 motif located on S3 as already suggested by Pedretti et al. [38]. Furthermore, SKF96365 exhibited strong interactions with Y745, while BCTC was unable to bind to this residue.

## Discussion

In light of the potential involvement of TRPM8 channels in the pathophysiology of cold nociception and cold allodynia [reviewed by [17]], there is a strong interest in the pursuit of novel modulators of TRPM8 channels. In this study, we investigated the effect of different compounds at the TRPM8-Y745H mutant channel, focusing on the differential effects of several antagonists on the gating properties.

Cold and menthol activate wild-type TRPM8 by shifting its voltage-dependent activation towards more negative potentials [3,6]. We confirmed a previous report showing that the tyrosine at position 745, on the putative S2 transmembrane segment, is essential for the agonist effects of menthol [35]. Menthol is a small and fairly lipophilic compound which easily partitions in the lipid bilayer and could thus be expected to affect TRPM8 channel function from various interaction sites. However, during menthol application to the menthol-insensitive TRPM8-Y745H mutant, the parameters describing voltage gating: V_1/2 _and g/g_ctrl _underwent no changes whatsoever, suggesting that menthol exerts its full effect by specific interaction with the binding site governed by the tyrosine residue 745. In the absence of menthol, the apparent gating charge, z_app_, was slightly lower in the TRPM8-Y745H mutant, indicating that this site is to some extent communicated with the voltage sensor of the channel. This finding is supported by recent work on TRPM8 voltage sensor mutants [39] and a fragmental model of the channel structure [38].

While TRPM8 and TRPV1 share only around 20% protein sequence homology [2], the channels have several phenotypic characteristics in common. Both are activated by temperature changes, and C-terminal chimeras between the channels exhibit reversed temperature sensitivity [40]. Furthermore, both TRPM8 and TRPV1 are inhibited by compounds such as BCTC, CTPC, SB-452533, capsazepine and ruthenium red [24,29]. All TRPV1 antagonists, with the exception of pore blockers such as ruthenium red, seem to bind at the vanilloid binding pocket [41]. However, no reports exist on the binding site of TRPM8 antagonists.

In this study, we demonstrate the variable inhibitory effect of several compounds at the TRPM8-Y745H mutant channel. This finding is unexpected since all of them affect gating of the channel in a similar fashion, shifting voltage-dependence to more positive potentials and exerting an allosteric negative modulation of the channel during cold- or chemically evoked activation [26]. Our results reveal that the tyrosine residue 745 at the menthol binding site is critical for inhibition mediated by SKF96365. In contrast, the inhibition by other antagonists is unaffected (BCTC) or only partially reduced (capsazepine, clotrimazole, econazole) by the mutation, suggesting that at least one other binding site exists on the TRPM8 channel from where the inhibitors exert their negative allosteric modulation. Furthermore, the results imply that not all TRPM8 blockers should be considered competitive antagonists of menthol, even though the allosteric inhibitory effect they mediate lies in competition with the activating effect of cooling agents [26].

The experimental observations of differential interaction of Y745 with the antagonists SKF96365 and BCTC were further confirmed by molecular docking studies. Interestingly, in these simulations SKF96365 exhibited strong interactions with both Y745 and an asparagine residue, N799, that also interacts with icilin [42]. Looking at Figure 8C, SKF96365 can be hypothesized to lock the S2 and S3 domains into a fixed position, thereby preventing conformational shifts of the S4 domain induced by menthol (or cooling) that would lead to channel activation. This idea is further supported by our finding that SKF96365 inhibits the TRPM8-wt current at 33°C, a condition in which the channel is only activated by voltage, whereas no effect is seen at the Y745H mutant. BCTC, in contrast, blocks both the wild-type and mutant channels in this condition with similar potency, indicating the presence of an alternative binding site. Overall, our data suggest a sequential model of TRPM8 gating where chemical modulators can favour (e.g. menthol) or hinder (e.g. BCTC or SKF96365) the energetics of subsequent channel opening by cold temperature or voltage, from different binding sites.

Our results provide direct clues for the identification of different structural motifs required for antagonist binding, aiding in the design of new candidate molecules for specific inhibition of TRPM8 channels. They also provide tools for current efforts to resolve the crystal structure of TRP channels [43-45]. Further mutagenesis work is required to identify the remaining binding site(s) of the various TRPM8 blockers. One potential site is formed by residues in the S2-S3 linker region, known to be important for the sensitivity to icilin [42]. Another plausible location is centred on residues within the TRP domain. This domain is known to be important in the energetics of channel opening, i.e. translating drug binding into channel opening [35,46,47]. In this regard, we consider a very powerful approach to subject random generated libraries of TRPM8 mutant channels [35] to the inhibitory actions of the different antagonists characterized in this study. In this way, one could obtain unbiased structural information on the action of different inhibitors.

Finally, we also studied for the first time the inhibitory capacity of two members of the imidazole family: the parent compound itself, and the antimycotic agent econazole, at the cooling-activated TRPM8 channel. Imidazole was unable to inhibit neither of the two TRPM8 constructs, while econazole, similarly to its relative clotrimazole, proved to be a potent antagonist of the wild-type channel. Both econazole and clotrimazole lost potency at the Y745H mutant channel. This identification of a novel TRPM8 antagonist prompts further screening of imidazole-based compounds in the quest for new TRPM8 blockers, and offers indications for the design of more potent derivatives.

## Conclusion

In this report, we identify, for the first time, structural elements on the TRPM8 protein that are critical for channel antagonism, and demonstrate an important difference in the way antagonists interact with the menthol binding site of TRPM8. The results of this study provide valuable information for solving the TRPM8 channel structure as well as for the future design of new, specific modulator compounds that could be useful in the treatment of cold-evoked pain.

## Methods

### Generation of point-mutant TRPM8-Y745H

The full length cDNA encoding mouse TRPM8 (NM_134252) in pcDNA5 (Invitrogen) was kindly provided by Dr. Ardem Patapoutian, (Scripps Research Institute, La Jolla, CA). The TRPM8-Y745H mutant was obtained by site-directed mutagenesis with the following primers: (for) 5'-CGTGGTCTTCCACATCGCC-3'; (rev) 5'-GGCGATGTGGAAGACCACG-3', and Pfu Turbo Taq DNA Polymerase (Roche).

### Cell culture and transfection

HEK293 cells were cultured in DMEM containing 10% of foetal bovine serum and antibiotics, and plated in 2 cm^2 ^wells at 400.000-500.000 cells/well. 20-24 h after plating, the cells were co-transfected with eGFP (0.3 μg/well) and TRPM8-wt or TRPM8-Y745H (2 μg/well) by incubating them with a solution containing the plasmid DNA and Lipofectamine 2000 (Invitrogen) for 5 hours. Subsequently, the cells were trypsinized and replated on poly-L-lysine-coated round coverslips (6 mm diameter). 20-48 h post-transfection, GFP(+) cells were selected for calcium imaging or electrophysiology experiments.

### Calcium imaging

Prior to experiment, HEK293 cells transfected with the TRPM8-wt/Y745H constructs were incubated with 5 μM Fura-2AM dissolved in the bath solution and 0.02% Pluronic (both from Invitrogen) for 40 min at 37°C in darkness. Fluorescence measurements were made with a Leica DM IRE2 inverted microscope fitted with a CCD camera (Imago QE Sensicam, TillPhotonics, Graefelfing, Germany). Fura-2 was excited at 340 nm and 380 nm with a Polychrome IV monochromator (Till Photonics) and the emitted fluorescence was filtered with a 510 nm longpass filter. Calibrated ratios were displayed online with Till Vision software v4.01 (TillPhotonics). The bath solution in calcium imaging experiments, from here on referred to as "control solution", contained (in mM): 140 NaCl, 3 KCl, 2.4 CaCl_2_, 1.3 MgCl_2_, 10 HEPES and 10 glucose, and was adjusted to a pH of 7.4 with NaOH.

### Electrophysiology

Whole-cell voltage-clamp recordings of TRPM8-wt/Y745H transfected HEK293 cells were performed simultaneously with temperature recordings. Current development was monitored at a holding potential of -60 mV. For information on voltage-dependent effects, 1.25-s-duration voltage ramps from -100 to +150 mV were delivered at desired time points. Standard patch-pipettes (3-5 MΩ) were made of borosilicate glass capillaries (Harvard Apparatus Ltd, UK), and contained (in mM): 150 NaCl, 3 MgCl_2_, 5 EGTA, 10 HEPES, (278 mOsm/kg; pH 7.2, adjusted with NaOH). During electrophysiological recordings, a divalent cation -free bath solution was used to minimize desensitization, which contained: 150 NaCl, 10 EDTA, 10 HEPES and 10 glucose (pH 7.4 with NaOH). The divalent-free solution was used in the preparation of all chemical modulator containing solutions. Current signals were recorded with an Axopatch 200B patch-clamp amplifier (Molecular Devices, Sunnyvale, CA, U.S.A.) applying series resistance compensation (60-90%). Stimulus delivery and data acquisition were performed using pClamp 9 software (Molecular Devices). Data were sampled at 5 kHz (ramps) and 1 kHz (timecourse) and filtered online at 2 kHz.

### Temperature stimulation

Coverslip pieces with cultured cells were placed in a microchamber and continuously perfused with solutions warmed at 33°C. The temperature was adjusted with a water-cooled Peltier device placed at the inlet of the chamber, and controlled by a feedback device. Cold-sensitivity was investigated with a temperature decrease to 18-20°C (Figure 1B).

### Chemical modulators

The chemical substances studied for their modulatory effect on the TRPM8 constructs were the cooling agent L-menthol (Scharlau, Spain), and the antagonists SKF96365 (1- [2-(4-methoxyphenyl)-2-[3-(4-methoxyphenyl)propoxy]ethyl-1H-imidazole; Tocris Bioscience, Bristol, UK); clotrimazole (1- [(2-chlorophenyl)diphenylmethyl]-1H-imidazole, Sigma), econazole (1- [2-[(4-chlorophenyl)methoxy]-2-(2,4-dichlorophenyl)ethyl]-1H-imidazole, Sigma), imidazole (1,3-diazacyclopenta-2,4-diene, Sigma), capsazepine (N- [2-(4-Chlorophenyl)ethyl]-1,3,4,5-tetrahydro-7,8-dihydroxy-2H-2-benzazepine-2-carbothioamide, Sigma); and BCTC (4- [3-chloro-pyridin-2-yl]-piperazine-1-carboxylic acid [4-tert-butyl-phenyl]-amide), which was a kind gift from Grünenthal GmbH Aachen, Germany. ACD/ChemSketch Freeware, version 8.0 (Advanced Chemistry Development, Inc., Toronto, ON, Canada, http://www.acdlabs.com) was used to draw the structures of the various modulators shown in Figure 3.

### Experimental protocols and interpretation of results

Experiments were always performed in parallel with cells expressing TRPM8-wt and TRPM8-Y745H to minimize any day-to-day variability in experimental conditions. To estimate the shifts in the voltage-dependence of activation of TRPM8-wt/Y745H in HEK293 cells, current-voltage (I-V) relationships from voltage ramps were fitted with an equation that combines a linear conductance multiplied by a Boltzmann activation term [48]:

where *g *is the maximum whole-cell conductance, *E*_*rev *_is the reversal potential, *V*_1/2 _is the potential for half maximal activation and *s *is the slope factor. For each cell, fitting was started by analyzing a condition exhibiting inward current. The value obtained for the parameter *E*_*rev *_at this condition was used when fitting I-V curves that lacked inward current.

### Molecular modelling

TRPM8-ligand complexes were built using the TRPM8 model constructed by Pedretti et al. [38]. Ligand structures were downloaded from the PubChem compound NCBI component database http://pubchem.ncbi.nlm.nih.gov/ and used with no additional optimization. The protein-ligand docking and the analysis of interactions was accomplished with Autodock [49] implemented in the general purpose molecular modelling software Yasara [50], and optimized with AMBER 99 force field [51]. Docking trials were optimized and clustered to remove redundancy and sorted by binding energy. Figures were drawn with Pymol (DeLano Scientific, Palo Alto, California, USA. http://www.pymol.org).

### Western blot

A TRPM8 antibody was generated in the laboratory. Briefly, whole serum anti-TRPM8 was obtained by rabbit immunization with a peptide corresponding to amino acids 1-10 of mouse TRPM8 (MSFEGARLSM) conjugated to keyhole limpet hemocyanin. For immunoblot analysis of TRPM8-wt/Y745H transfected HEK293 cells, 48-h post transfection, cells were mixed with sample buffer (4% SDS (w/v); 10% glycerol (v/v); 4% 2-mercaptoethanol; 0.2% bromophenol blue (w/v); 50 mM Tris-HCl; pH 6.8), heated at 95°C for 5 min, and then subjected to SDS-PAGE gel electrophoresis (10% acrylamide gel). Proteins were transferred onto a nitro-cellulose membrane Hybond™ ECL (Amersham Biosciences). Membranes were blocked with 10% non-fat milk in TBS (Tris-buffered saline), and subsequently incubated with the primary antibody for TRPM8 at 1:500 dilution for 1 h at room temperature and at 4°C overnight. Blots were treated with peroxidase-conjugated goat anti-rabbit IgG at 1:2000 dilution for 1 h, and protein signals were revealed using the ECL Advance™ Western Blotting Detection Kit (Amersham Biosciences) and visualized with the LAS-1000 imaging system by Fujifilm.

### Data analysis

Data are reported as mean ± standard error of the mean. All fitting was carried out with the Levenberg-Marquardt method implemented in the Origin 7.0 software. In the dose-inhibition curves shown in Figure 5, error bars were used as weights in fits to the Hill equation. When comparing two means, statistical significance (p < 0.05) was assessed by Student's two-tailed *t*-test. For multiple comparisons of means, one-way or two-way ANOVA was performed using GraphPad Prism version 4.00 for Windows (GraphPad Software, San Diego California USA, http://www.graphpad.com). Paired or repeated-measures tests were used where appropriate to account for inter-individual variability.

## Competing interests

The authors declare that they have no competing interests.

## Authors' contributions

AM designed and carried out the electrophysiological studies, the statistical analyses, and drafted the manuscript. MP generated the point-mutant TRPM8-Y745H; conducted the calcium imaging studies and their analysis; as well as the immunoassays. GFB carried out the molecular modelling. AFM participated in the design of the molecular modelling and helped to draft the manuscript. FV conceived of the study, and participated in its design and coordination and helped to draft the manuscript. All authors read and approved the final manuscript.
